# Most outdoor malaria transmission by behaviourally-resistant *Anopheles arabiensis* is mediated by mosquitoes that have previously been inside houses

**DOI:** 10.1186/s12936-016-1280-z

**Published:** 2016-04-19

**Authors:** Gerry F. Killeen, Nicodem J. Govella, Dickson W. Lwetoijera, Fredros O. Okumu

**Affiliations:** Environmental Health and Ecological Sciences Thematic Group, Ifakara Health Institute, Ifakara, Kilombero, Morogoro, United Republic of Tanzania; Department of Vector Biology, Liverpool School of Tropical Medicine, Liverpool, UK; School of Public Health, University of the Witwatersrand, Johannesburg, Republic of South Africa

**Keywords:** *Plasmodium*, Malaria, *Anopheles*, Mosquito, Vector control, Residual transmission

## Abstract

**Background:**

*Anopheles arabiensis* is stereotypical of diverse vectors that mediate residual malaria transmission globally, because it can feed outdoors upon humans or cattle, or enter but then rapidly exit houses without fatal exposure to insecticidal nets or sprays.

**Methods:**

Life histories of a well-characterized *An. arabiensis* population were simulated with a simple but process-explicit deterministic model and relevance to other vectors examined through sensitivity analysis.

**Results:**

Where most humans use bed nets, two thirds of *An. arabiensis* blood feeds and half of malaria transmission events were estimated to occur outdoors. However, it was also estimated that most successful feeds and almost all (>98 %) transmission events are preceded by unsuccessful attempts to attack humans indoors. The estimated proportion of vector blood meals ultimately obtained from humans indoors is dramatically attenuated by availability of alternative hosts, or partial ability to attack humans outdoors. However, the estimated proportion of mosquitoes old enough to transmit malaria, and which have previously entered a house at least once, is far less sensitive to both variables. For vectors with similarly modest preference for cattle over humans and similar ability to evade fatal indoor insecticide exposure once indoors, >80 % of predicted feeding events by mosquitoes old enough to transmit malaria are preceded by at least one house entry event, so long as ≥40 % of attempts to attack humans occur indoors and humans outnumber cattle ≥4-fold.

**Conclusions:**

While the exact numerical results predicted by such a simple deterministic model should be considered only approximate and illustrative, the derived conclusions are remarkably insensitive to substantive deviations from the input parameter values measured for this particular *An. arabiensis* population. This life-history analysis, therefore, identifies a clear, broadly-important opportunity for more effective suppression of residual malaria transmission by *An. arabiensis* in Africa and other important vectors of residual transmission across the tropics. Improved control of predominantly outdoor residual transmission by *An. arabiensis*, and other modestly zoophagic vectors like *Anopheles darlingi*, which frequently enter but then rapidly exit from houses, may be readily achieved by improving existing technology for killing mosquitoes indoors.

## Background

**Fig. 1:**
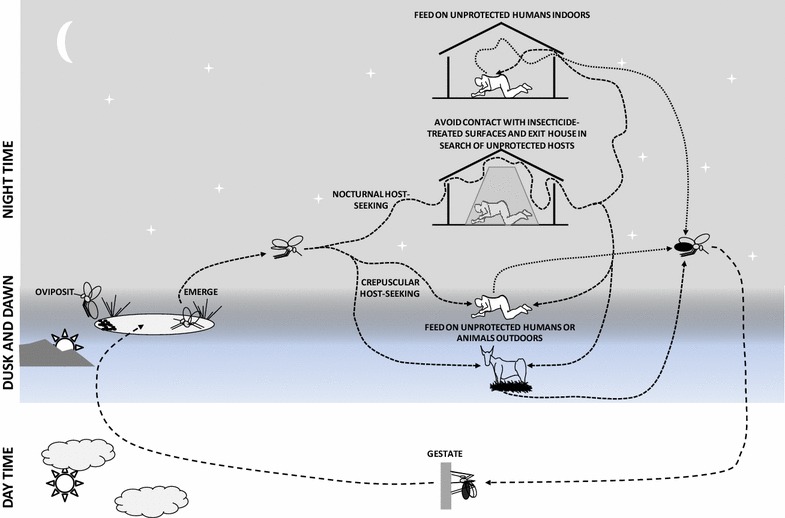
A schematic summary of how specific behaviours enable mosquito populations generally, or *Anopheles arabiensis* in southern Tanzania specifically [16–19], to survive and mediate residual malaria transmission, despite high usage rates of long-lasting insecticidal nets

The World Health Organization (WHO) has recently acknowledged that *residual* malaria transmission can persist despite comprehensive, population-wide coverage of long-lasting insecticidal nets (LLINs) and indoor residual spraying (IRS) with active ingredients to which local vector populations are fully susceptible [1, 2]. Residual transmission occurs because vector mosquitoes exhibit one or more behaviours that allow them to evade fatal contact with these front line interventions (Fig. 1) [1–4]. Feeding upon humans when they are unprotected outdoors is the most obvious of these behaviours [3–8], but vectors may also survive and mediate residual malaria transmission despite high coverage with LLINs and/or IRS by feeding upon animals instead [4, 9–12]. While most of these behaviours have always existed naturally in vector populations that can therefore be described as *resilient* [13], it also appears that heritably modified behaviours have been selected for by widespread use of LLINs and IRS, resulting in vector populations that can be described as behaviourally *resistant* in the strict sense [14]. Regardless of whether these behaviours represent resilience or resistance, elimination of malaria transmission from many endemic settings will require new or improved anti-vector measures that target mosquitoes when they feed outdoors upon humans or livestock, or at source in the aquatic habitats their immature stages develop in [1, 4].

However, a more subtle behavioural phenotype that allowed *Anopheles darlingi*, *Anopheles nunetzovari* and *Anopheles punctimacula* to survive and sustain malaria transmission in Latin America during the Global Malaria Eradication Programme [15], and allows *Anopheles arabiensis* to sustain residual transmission across the Rift Valley region of east Africa today [16–19], is the ability to enter houses protected with LLINs and IRS but then leave again rapidly before lethal insecticide exposure can occur. Indeed, it is striking that despite the scale up of IRS and LLINs in recent years, several African vector populations have maintained strong preferences for feeding at night when humans are asleep indoors [20–22]. Simulation analysis confirms that this phenotypically plastic ability to evade lethal contact with insecticide-treated surfaces once inside a house, allowing individual mosquitoes to instantaneously alter their behaviours in response to changes in their environment [13], has substantial fitness advantages over fixed, innate, inherited preferences for feeding at dawn and dusk, when humans are active and protected by their bed nets [14].

*Anopheles arabiensis* represents a particularly interesting example of a behaviourally resilient or resistant malaria vector, because it is widespread across most of Africa [23] and exhibits all three of the behaviours thought to facilitate residual transmission: (1) it may feed upon humans outdoors at dawn and dusk when they are outside of their protected homes and sleeping spaces [24–26]; (2) it readily feeds upon cattle where they are available [27, 28]; (3) it may enter and exit houses protected with IRS or LLINs, but then leave again safely without fatal exposure to insecticide-treated surfaces (Fig. 1) [3, 16–19, 29, 30]. The *An. arabiensis* population of the Kilombero Valley in southern Tanzania is particularly well characterized and has maintained stable, remarkably intense transmission of *Plasmodium falciparum* despite high usage rates of LLINs over several years, even before [31] the emergence of wide-spread pyrethroid resistance in the area [32]. Immediately after the transition from high usage rates of untreated bed nets to high usage of LLINs and much lower transmission intensity in 2008 [31], altered biting times patterns were observed, so that approximately one fifth of exposure to this mosquito occurred outdoors for residents lacking a protective net (*π*_*h,o,0*_ = 1 − *π*_*h,i,0*_ = 18 %; see Table 1 for parameter notation definitions) [25]. Furthermore, because their indoor exposure was reduced by personal protection, more than three quarters of remaining exposure occurred outdoors for net users (*π*_*h,o,n*_ = 1 − *π*_*h,i,n*_ = 77 %, with *π*_*h,i,n*_ calculated as a function of *π*_*h,i,0*_ and protective efficacy of bed nets at times when they are actually used (*ρ*); see Table 1 for parameter notation definitions) [33].

**Table 1:** Model parameter symbols and definitions

Symbol	Definition
*Parameters*	
*E*_*N*_	Mean number of encounters required for a mosquito to feed each at the end of each host-seeking interval, in a scenario with high usage of bed nets (Ω = *N*)
*F*_*x,N*_	Proportion of mosquitoes that have been inside house but failed to feed because the occupants were protected by LLINs on at least one occasion by the time they had completed their *x* ^th^ blood meal, in a scenario with high usage of bed nets (Ω = *N*)
*I*_*c*_	Number of individual cattle (*c*) present
*I*_*h*_	Number of individual humans (*h*) present
*λ*_*c*_	Relative availability of blood from an individual head of cattle to vector mosquitoes, compared to an individual human
*Ω*	Vector control intervention scenario with either high usage of bed nets (*Ω* = *N*) or no bed net use (*Ω* = *0*)
*ρ*	Proportional protective efficacy of using a bed net against biting vectors at times when the bed net is actually used
*π*_*h,i,0*_	Proportion of vector biting exposure that occurs indoors (*i*) for human (*h*) individuals or populations not using bed nets (*0*)
*π*_*h,i,n*_	Proportion of vector biting exposure that occurs indoors (*i*) for human (*h*) individuals using bed nets (*n*)
*π*_*h,i,N*_	Average proportion of vector biting exposure that occurs indoors (*i*) across an entire human (*h*) population with high usage of bed nets (Ω = *N*)
*π*_*h,o,N*_	Average proportion of vector biting exposure that occurs outdoors (*o*) across an entire human (*h*) population with high usage of bed nets (Ω = *N*)
*Q*_*h,N*_	Proportion of vector blood meals obtained from humans in a scenario with high usage of bed nets (Ω = *N*)
*Q*_*h,i,N*_	Proportion of vector blood meals obtained from humans while indoors in a scenario with high usage of bed nets (Ω = *N*)
*Q*_*h,o,N*_	Proportion of vector blood meals obtained from humans while outdoors in a scenario with high usage of bed nets (Ω = *N*)
*Q*_*c,N*_	Proportion of vector blood meals obtained from cattle in a scenario with high human usage of bed nets (Ω = *N*)
*Z*_*h,i,N*_	Availability of all indoor human blood sources to vectors, expressed as the mean rate at which mosquitoes find, attack and successfully feed upon all humans (*h*) at times when they are indoors (*i*), in a scenario with high usage of bed nets (Ω = *N*)
*Z*_*h,N*_	Availability of all human blood sources to vectors, expressed as the mean rate at which mosquitoes find, attack and successfully feed upon all humans (h) at any time, in a scenario with high usage of bed nets (Ω = *N*)
*Z*_*c,N*_	Availability of all cattle blood sources to vectors, expressed as the mean rate at which mosquitoes find, attack and successfully feed upon all cattle (c) at any time, in a scenario with high usage of bed nets (Ω = *N*)
*Subscripts*	
*c*	Cattle
*h*	Humans
0	No net use by a human individual or population
*n*	Bed net use by an individual human
*N*	Scenario (*Ω*) with high rates of bed net use (*U*_*h*_ = 0.8) by an entire human population
*x*	Number of feeding cycles completed by a mosquito or age cohort in a mosquito population

Furthermore, more than half of all blood meals by this species were obtained from cattle during the same period (*Q*_*c,N*_ = 51 %; see Table 1 for parameter notation definitions) [34], presumably because increased livestock-rearing activities have augmented the availability of bovine blood while net use has made human blood, which used to account most *An. arabiensis* blood meals [35], increasingly difficult for mosquitoes to access during their preferred nocturnal feeding times. However, perhaps the most worrying observation from this setting is that, even when *An. arabiensis* enter houses to attack humans, no combination of LLINs and IRS, regardless of the products or combinations of active ingredients used, kills more than a quarter of them before they leave in search of unprotected victims elsewhere [17, 18]. Because it exhibits all three of the major behavioural phenotypes that allow mosquitoes to evade fatal exposure to LLINs and IRS (Fig. 1), the *An. arabiensis* population in this part of southern Tanzania may be described as a stereotype of all the dozens of vectors that mediate residual malaria transmission globally.

The latter of these three behavioural traits may confer significant fitness advantages where LLINs are applied at high coverage because extended but cautious foraging throughout the night allows mosquitoes far more opportunities to feed upon cattle or humans lacking nets than restricting feeding only to brief periods when nets are not used [14]. Such a cautious, evasive approach to protected humans inevitably implies that foraging activity must be extended to eventually obtain blood, so that greater numbers of frustrated host encounters must occur before mosquitoes can finally feed successfully [14]. Here a simple process-explicit model is applied to estimate what proportion of blood meals these *An. arabiensis* obtain from humans indoors, humans outdoors and from cattle, and then the proportion of feeding and malaria transmission events that are preceded by unsuccessful encounters with humans inside houses while foraging. This life-history analysis reveals that, regardless of where transmission ultimately occurs, most of the older mosquitoes mediating it have previously been inside at least one house, where they could have been killed with one of the rapidly emerging new methods for improved indoor vector control.

## Methods

While essentially identical results were also obtained by using far more complex, detailed models of mosquito behaviour and malaria transmission [14, 17, 36], a far simpler form of these models [37] is applied here to illustrate key processes at hand as clearly as possible to a broad epidemiological audience.

A simple kinetic model of mosquito foraging for blood [37] is extended slightly, to distinguish between the availability of blood hosts to encounter and attempt attack upon (*A*) and the availability of blood per se (*Z*) [36]. Note that both of these distinctive availability terms are defined as the total rates at which the relevant attack (*A*) or feeding (*Z*) events occur on all hosts, or a specified subset of hosts, per individual foraging, host-seeking mosquito [36]. Note that this model assumes a single, homogenously-mixed mosquito population that is equally likely to attempt to attack humans when they are encountered indoors or outdoors, but that the distribution of human exposure across indoor and outdoor spaces is determined by where and when human and mosquito activities overlap [14, 20]. All cattle are assumed to always be outdoors, but this has no impact upon the model because they are also assumed to be all unprotected and equally vulnerable to attack indoors and outdoors. All parameter definitions and symbols are detailed in Table 1.

Assuming the same input values for baseline human (*Z*_*h,0*_) and cattle (*Z*_*c,0*_) host availability as recent simulations of the impacts of LLINs and IRS combinations upon *An. arabiensis* in the Kilombero Valley [17], there are assumed to be 140 cattle for every 1000 humans (*I*_*c*_*/I*_*h*_ = 0.14) and each head of cattle is assumed to have 61 % greater availability to foraging mosquitoes than an average human (*λ*_*c*_ = 1.61) [38], presumably because of their larger size and greater attractiveness to this vector species. To enable simplification of the equations for proportional output parameters, specifically the proportion of all blood meals obtained from cattle (*Q*_*c*_) or human (*Q*_*h*_) host subsets and the proportions of human biting exposure occurring indoors (*π*_*h,i*_) or outdoors (*π*_*h,o*_), all blood availability terms for cattle (*Z*_*c*_) and protected human populations (*Z*_*h,N*_, *Z*_*h,i,N*_ and *Z*_*h,o,N*_) are calculated relative to *Z*_*h,0*_. The proportion of exposure to *An. arabiensis* occurring indoors for unprotected humans lacking a bed net (*π*_*h,i*_) is assumed to match field measurements of 82 % [25], representing the de facto limit of the level of personal protection that even a net with very high protective efficacy (*ρ* = 90 %) [39] can provide. Assuming that cattle and humans are the only major sources of blood for this vector [38], the proportions of blood meals obtained from humans, protected or otherwise (*Q*_*h*_), and cattle (*Q*_*c*_) is equivalent to the proportion of all available sources of blood that these host species account for (*Z*_*h*_ and *Z*_*c*_, respectively). For a scenario with no LLINs (*Ω* = 0), these human and cattle blood indices can be predicted (*Q*_*h,0*_ and *Q*_*c,0*_) as a simple function of the relative population sizes and relative availability of individuals from these two host populations as sources of blood (*λ*_*c*_ for one head of cattle relative one human):

1 $$Q_{h,0} = \frac{{Z_{h,0} }}{{Z_{h,0} + Z_{c} }} = \frac{1}{{1 + \lambda_{c} (I_{c} /I_{h} )}}$$

2 $$Q_{c,0} = 1 - Q_{h,0} = \frac{{Z_{c} }}{{Z_{h,0} + Z_{c} }} = \frac{{\lambda_{c} (I_{c} /I_{h} )}}{{1 + \lambda_{c} (I_{c} /I_{h} )}}$$

Furthermore, in the absence of bed nets (*Ω* = 0) the proportion of all blood meals obtained from humans while indoors (*Q*_*h,i,0*_) and outdoors (*Q*_*h,o,0*_) can be separately calculated based on the measured proportion of human exposure that occurs indoors (*π*_*h,i,0*_) and outdoors (*π*_*h,o,0*_ = 1 − *π*_*h,i,0*_):

3 $$Q_{h,i,0} = \frac{{Z_{h,i,0} }}{{Z_{h,0} + Z_{c} }} = \frac{{\pi_{h,i,0} }}{{1 + \lambda_{c} (I_{c} /I_{h} )}}$$

4 $$Q_{h,o,0} = \frac{{Z_{h,o,0} }}{{Z_{h,0} + Z_{c} }} = \frac{{1 - \pi_{h,i,0} }}{{1 + \lambda_{c} (I_{c} /I_{h} )}}$$

Predictions of these same proportional contributions of various blood sources (*Q*_*h,N*_, *Q*_*h,i,N*_*, Q*_*h,o,N*_ and *Q*_*c,N*_) can also be made for a scenario with high bed net use (*Ω* = *N)*, specified as four fifths of residents using bed nets (*U*_*h*_ = 80 %). The availability of human blood is reduced in proportion to the *de facto* mean level of personal protection provided by those nets at population level when limitations of human net usage rates (*U*_*h*_) and the proportion of bites that would otherwise occur indoors (*π*_*h,i,0*_) are accounted for:

5 $$Q_{h,N} = \frac{{Z_{h,N} }}{{Z_{h,N} + Z_{c} }} = \frac{{1 - \rho \pi_{h,i,0} U_{h} }}{{(1 - \rho \pi_{h,i,0} U_{h} ) + \lambda_{c} (I_{c} /I_{h} )}}$$

6 $$Q_{c,N} = 1 - Q_{h,N} = \frac{{Z_{c} }}{{Z_{c} + Z_{h,N} }} = \frac{{\lambda_{c} (I_{c} /I_{h} )}}{{(1 - \rho \pi_{h,i,0} U_{h} ) + \lambda_{c} (I_{c} /I_{h} )}}$$

7 $$Q_{h,i,N} = \frac{{Z_{h,i,N} }}{{Z_{h,N} + Z_{c} }} = \frac{{\pi_{h,i,0} (1 - \rho U_{h} )}}{{(1 - \rho \pi_{h,i,0} U_{h} ) + \lambda_{c} (I_{c} /I_{h} )}}$$

8 $$Q_{h,o,N} = \frac{{Z_{h,o,N} }}{{Z_{h,N} + Z_{c} }} = \frac{{1 - \pi_{h,i,0} }}{{(1 - \rho \pi_{h,i,0} U_{h} ) + \lambda_{c} (I_{c} /I_{h} )}}$$

The proportion of all human biting exposure that occurs outdoors can then be readily estimated as the outdoor fraction of all blood meals obtained from humans:

9 $$\pi_{h,o,N} = \frac{{Z_{h,o,N} }}{{Z_{h,N} }} = \frac{{1 - \pi_{h,i,0} }}{{1 - \rho \pi_{h,i,0} U_{h} }}$$

Total blood availability is defined as the rate at which individual mosquitoes encounter, attack and successfully feed upon all hosts, so it is inversely proportional to the length of time spent foraging and number of host encounters required to do so [36]. So, for example, if high coverage with protective measures like bed nets prevent successful feeding following half all host encounter events, total blood availability will be halved and each successful blood meal will require twice as many host encounters. Assuming that essentially all encounters with hosts in the absence of nets result in a successful feeding event, the mean number of encounters required for a mosquito to feed each at the end of each host-seeking interval in the presence of nets (*E*_*N*_) can be estimated as the total blood availability in the absence of nets (*Z*_*0*_), divided by the total blood availability in the presence of nets (*Z*_*N*_):

10 $$E_{N} = \frac{{Z_{0} }}{{Z_{N} }} = \frac{{1 + \lambda_{c} (I_{c} /I_{h} )}}{{(1 - \rho \pi_{i} U_{h} ) + \lambda_{c} (I_{c} /I_{h} )}}$$

Obviously, the longer a mosquito lives and has to spend foraging for hosts, the greater the number of host encounters it experiences, and the lower the probability that it has never been inside a house. The proportion of mosquitoes that have been inside a house at least once therefore increases to saturation with age, at a rate that increases as protective measures like nets necessitate more host encounters per successful blood meal. The proportion of mosquitoes that have been inside house but failed to feed because the occupants were protected by LLINs at least once (*F*_*N*_), can therefore be calculated as an exponential decay function of the number of failed encounters per gonotropic cycle (*E*_*N*_*−*1) and the age of the mosquito in terms of the number of gonotrophic cycles (*x*) it has completed:

11 $$F_{x,N} = 1 - e^{{ - x (E_{N} - 1)}}$$

Note that while insecticide treatment of nets causes negligible additional mortality of *An. arabiensis* in this setting [18] and another recently described setting in Ethiopia [19], this phenomenon does need to be assumed because this model only predicts the choices of those mosquitoes that actually fed successfully and, therefore, survived any hazards associated with foraging and all attempted host attacks. The parameters for mean proportional bed net usage (*U*_*h*_) and mean protective efficacy while in use (*ρ*) therefore represent those for all treated and untreated nets present in the community.

## Results

Similar to field observations from the Kilombero Valley [34], the model predicts that more than a third of all blood meals are obtained from cattle where nets are widely used (*Q*_*c,N*_ = 37 %). The proportion of blood meals obtained from humans reported by this case–control field study was somewhat higher than those simulated here because that field study design specified that half of the households selected for inclusion owned cattle, whereas the highest level of mean household cattle ownership in any of the 10 study villages was only 16 % [34]. The model predictions indicate that the remaining two thirds of all blood meals were obtained from humans, distributed approximately evenly across indoor (*Q*_*h,i,N*_ = 33 %) and outdoor (*Q*_*h,i,N*_ = 30 %) biting exposure events. However, unlike previous modelling investigations, which only considered the times and places where mosquitoes ultimately feed upon humans as possible opportunities to kill them [24], this simulation analysis examines the life history of the overall host-seeking process and suggests even greater reason for optimism.

Regardless of where *An. arabiensis* ultimately feeds and mediates malaria transmission, these simulations predict that most mosquitoes which do eventually feed have previously been inside an occupied house: given the input parameters described above, each successful blood meal in the presence of nets is preceded by an average of one unsuccessful encounter (*E*_*N*_ = 2.01 host encounters per completed gonotrophic cycle) with a human net user inside a house, forcing the mosquito had to leave again and keep foraging until it found an unprotected human or bovine victim. Overall, it is estimated that almost two thirds of all successfully fed mosquitoes, including those that fed outdoors upon humans or cattle, had previously entered a house but failed to feed on its protected human occupants, at least once during the same foraging process (*F*_*x,N*_ = 63 % where *x* = 1 blood meal). Furthermore, by the time mosquitoes live long enough to transmit malaria, almost all of them are predicted to have previously been inside a house but failed to feed upon the net users sleeping inside (*F*_*x,N*_ = 98 % where *x* = 4 blood meals, the minimum number of gonotrophic cycles of 3 days mean duration that is required to enable transmission where the sporogonic incubation for the parasite period is 10 days).

**Fig. 2:**
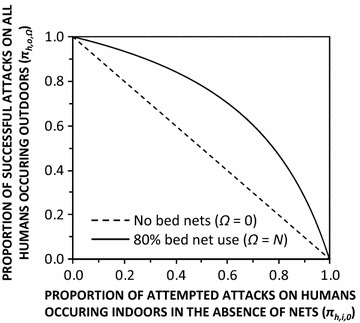
Dependence of the population-wide mean proportion of human exposure occurring outdoors with (*Ω* = *N*) and without (*Ω* = *0*) 80 % bed net usage upon the proportion occurring indoors in the absence of any nets, for a vector population with the same ability as *Anopheles arabiensis* to avoid fatal contact with LLINs or IRS after entering houses [16–19], so that the insecticide treatment status of the net is irrelevant

Of course *An. arabiensis* populations, with these particular behavioural parameters in this well-studied part Tanzania, are merely a motivating example of an understudied phenomenon that has also been described among several important malaria vectors of Latin America, and may be widespread amongst a variety of *Anopheles* populations across the tropics [14]. Applying sensitivity analysis to the same model illustrates how remarkably high proportions of residual human exposure to biting vectors can occur outdoors when nets are widely used, even in settings where the vector has a strong preference for feeding indoors (Fig. 2). Obviously, where a vector preferentially attempts to attack humans almost exclusively indoors (*π*_*h,i,N*_ = ≥90 %), most transmission will still occur indoors even if high LLINs coverage is achieved. However, this relationship is very sensitive to even slight shifts towards preference for outdoor biting, so outdoor biting represents a considerable proportion of residual human exposure following LLIN scale up where vectors have even a partial ability to feed outdoors (Fig. 2). If even as little as a quarter of exposure of residents lacking a net occurs outdoors (*π*_*h,o,0*_ = 25 %), slightly more than half of all exposure across the entire human population is expected to occur outdoors (*π*_*h,o,N*_ = 54 %). Where human exposure in the absence of nets is equally likely to occur indoors or outdoors (*π*_*h,o,0*_ = *π*_*h,i,0*_ = 50 %), more than three quarters of residual transmission is predicted to occur outdoors when nets are used at high coverage (*π*_*h,o,N*_ = 78 %).

**Fig. 3:**
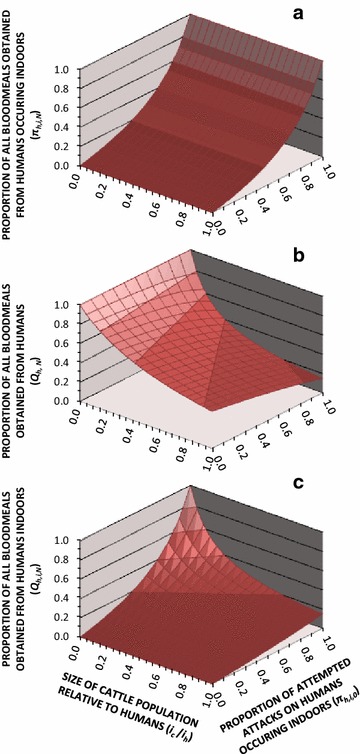
The influence of varying levels of cattle availability and baseline distribution of human biting exposure indoors and outdoors upon the predicted proportions of all human blood meals that are obtained indoors (**a**), all blood meals obtained from humans (**b**), and all blood meals obtained from humans indoors (**c**). The predictions presented are based on simulations assuming a setting with high bed net usage (*Ω* = *N*) and vector mosquitoes have the same ability as *Anopheles arabiensis* to avoid fatal contact with LLINs or IRS after entering houses [16–19], so that the insecticide treatment status of the net is irrelevant

Proportional distribution of human exposure to residual transmission across indoor and outdoor locations in the presence of LLINs is predicted to vary with the corresponding distribution in their absence, regardless of how many alternative hosts are present (Fig. 3a). However, the proportion of blood meals obtained from humans and, therefore, the proportion of transmission events that can be directly interrupted by preventing human exposure, is almost as sensitive to increasing availability of alternative blood sources, such as cattle, as it is to the propensity of the vector to feed outdoors rather than indoors (Fig. 3b). Considering both of these factors, less than half of all blood meals are obtained from humans indoors at high bed net coverage (*Q*_*h,i,N*_ < 0.5) unless the cattle populations are sparse (*N*_*c*_/*N*_*h*_ < 0.1) and the vector almost exclusively attempts to attack humans indoors in the absence of bed nets (*π*_*h,i,0*_ ≥ 90 %) (Fig. 3c).

If it is assumed that only the successful feeding event itself can be targeted with vector control measures, these simulations suggest that more effective control, and certainly, elimination of residual malaria transmission necessitates interventions targeted at outdoor attempts to attack humans and livestock [1, 3, 12]. However, the analysis of where human exposure eventually occurs, and where the vectors ultimately obtain blood, merely represents a readily-observed endpoint of successful host seeking processes. Examining the simulated life histories of the fraction of the vector population that does successfully feed illustrates vulnerabilities that may be exploited with improved vector control measures targeted at human housing. Specifically, it illustrates how improved interventions for killing mosquitoes indoors could effectively target an epidemiologically significant proportion of residual transmission, even where the vectors often feed opportunistically upon humans or cattle outdoors.

**Fig. 4:**
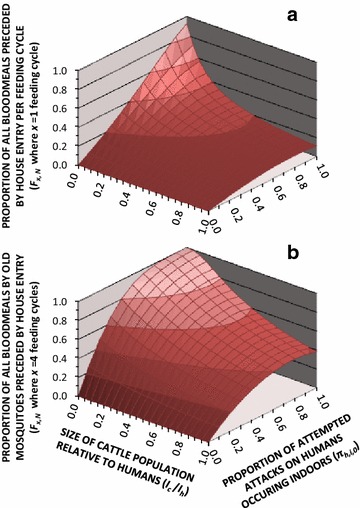
The influence of varying levels of cattle availability and baseline distribution of human biting exposure indoors and outdoors upon the predicted proportions of all mosquitoes successfully obtaining a blood meal which had previously entered but then left a house unfed in a single feeding cycle (**a**) or the minimum number of feeding cycles required to acquire and transmit *Plasmodium falciparum* malaria (**b**). The predictions presented are based on simulations assuming a setting with high bed net usage (*Ω* = *N*) and vector mosquitoes have the same ability as *Anopheles arabiensis* to avoid fatal contact with LLINs or IRS after entering houses [16–19], so that the insecticide treatment status of the net is irrelevant

While the proportion of all blood meals ultimately obtained from humans indoors is dramatically attenuated by the availability of alternative hosts or even partial ability to attack humans outdoors where LLINs are widely used, the probability of having entered a house before successfully feeding is less sensitive to these behavioural determinants (Fig. 4a), especially if calculated cumulatively until the mosquito has lived long enough to acquire and transmit malaria (Fig. 4b). For vector species with the same ability as *An. arabiensis* to evade fatal exposure to LLINs and IRS inside houses, and the same preference for cattle over humans, at least 80 % of simulated feeding events by mosquitoes old enough to transmit malaria (*F*_*x,N*_ >80 %, where *x* = 4) are preceded by a house entry event, so long as at least 40 % of human exposure occurs indoors for non-users of bed nets and humans outnumber cattle by at least 4 to 1.

## Discussion

The greatest strength and limitation of this analysis is the simple, deterministic nature of the model used. The obvious limitation of this parsimonious approach is that additional subtleties, such as cumulative exposure to insecticides over multiple house entry events and phenomena arising as exceptions from stochastic processes, may not be explored. However, it does have the advantage of being relatively accessible to biologists and epidemiologists lacking specialist mathematics expertise. While the exact numerical results predicted by such a simple deterministic model should be considered only approximate and illustrative, the derived conclusions are remarkably insensitive to substantive deviations from the input parameter values measured for this particular *An. arabiensis* population. These simulated life history summaries are sufficient to identify clear and attractive opportunities for effectively controlling malaria residual transmission, mediated both indoors and outdoors by vectors like *An. arabiensis*, by simply improving existing technologies for killing mosquitoes indoors. In other words, new strategies for controlling predominantly outdoor residual transmission that persists following scale up of indoor bed net use, do not necessarily have to target the responsible vector outdoors, but could also target them indoors wherever they commonly make unsuccessful indoor foraging attempts before eventually feeding upon unprotected cattle or humans outdoors.

Whether these conclusions apply to other vectors that also forage persistently but cautiously for humans inside houses [15], remains to be seen. On the one hand, at least partial endophagy appears the rule rather than the exception: No vector population for which estimates are available encounters and attempts to attack humans indoors on less than 40 % of occasions [4]. On the other hand, strong preference for non-human hosts appears to be the rule rather than the exception amongst malaria vectors, although it should be noted that this trait also makes vectors far less efficient [4]. *Anopheles arabiensis* has only a modest preference for cattle over humans [38], and humans outnumber cattle in most African settings, including this specific example of the Kilombero Valley in Tanzania. Except in settings with unusually high population ratios of cattle to humans, the latter can remain an important source of nutrition for this species, even after high protective coverage with bed nets is achieved. Supplementary vector control measures targeting mosquitoes when they attack people may therefore achieve considerable incremental impact upon residual malaria transmission.

However, most of the world’s vectors are probably too zoophagic [4] to be effectively tackled with purely indoor control measures, even assuming that they exhibit the same kind of early-exit behaviours as *An. arabiensis* when they do enter houses (Fig. 4b). Low-intensity malaria transmission mediated by the plethora of highly zoophagic vector species distributed ubiquitously across the tropics should be particularly responsive to personal protection measures, such as vapour-phase insecticides [40], regardless of whether these kill or merely repel mosquitoes [9, 10, 12]. For such species with so little dependence upon human blood for their survival [9, 10], mass effects through vector population suppression will probably require veterinary insecticides targeted at preferred livestock species [4, 12, 41]. One potentially important exception is *An. darlingi*, the most important malaria vector in the Americas, which shares several behavioural commonalities with *An. arabiensis* in Africa. *A. darlingi* typically obtains half of its blood meals from humans [42], usually attempts to attack them indoors if they lack protective bed nets [4], and was one of the first incriminated vectors of residual transmission shown to exit from houses within an hour and to rest on insecticide-sprayed surfaces for only 1 min [15].

One controversial way to tackle such vectors, which is practiced in several countries today, is to allow them to feed by applying IRS without LLINs, so that they are killed when they rest after feeding: IRS with the organophosphate pirimiphos methyl kills high proportions of house-entering *An. arabiensis* in the absence of nets [43] but not when they are used [18]. However, such a strategy requires human exposure to feeding vector mosquitoes to keep them in houses for long enough to kill them, raising obvious concerns about practical and ethical acceptability [36, 44].

Fortunately, it also appears possible to dramatically increase their mortality rates inside houses without relying on continued human exposure, by physically retaining them inside treated structures with baffled entry points treated with chemical [45] or biological [46] insecticides to maximize exposure when they attempt to exit via the same route [14]. A key advantage of the entomopathogenic fungi used in the latter studies is their ability to grow into a lethal dose following even the briefest exposure of evasive mosquitoes like *An. arabiensis* [46].

Furthermore, recently evaluated electrostatic coatings, now enable enhanced and accelerated transfer of more traditional chemical insecticides to mosquitoes making similarly brief contacts with netting materials [47]. It has also recently been demonstrated that existing, WHO-recommended insecticide formulations for conventional IRS, can be used against *An. arabiensis* with improved effect, by applying them to netting baffles placed over the eave gaps of experimental huts (Killeen et al. Unpublished). Perhaps most exciting of all is a recently-developed device lacking any insecticide, which kills mosquitoes by simply trapping them when they attempt to enter houses [48]. Taking these prototypes as examples of what is possible with remarkably simple, low-technology innovations, it should feasible to rapidly and substantially enhance control of residual malaria transmission mediated indoors and outdoors by vectors like *An. arabiensis* and *An. darlingi*, by simply optimizing combinations of existing insecticides with novel delivery formats for killing them when they enter houses.

## Conclusions

New technologies for protecting humans while active outdoors, or for killing mosquitoes feeding upon livestock, will probably be needed to eliminate residual malaria transmission by vector species feeding predominantly upon animals [4, 10, 12, 41]. However, in many settings where widespread bed net use forces malaria vectors to predominantly feed outdoors, the life histories of mosquitoes old enough to transmit malaria usually include at least one attempt to attack a human indoors. Improved control of outdoor transmission by *An. arabiensis*, and other modestly zoophagic vectors which, like *An. darlingi*, frequently enter but rapidly exit from human habitations, may therefore also be achieved with simple improvements to existing technology for killing mosquitoes inside houses.
